# Utilizing the Un-Meeting model to advance innovative translational and team science

**DOI:** 10.1017/cts.2023.576

**Published:** 2023-07-10

**Authors:** Erika F. Augustine, Scott J. Steele, Scott McIntosh, Laura Sugarwala, Robert J. White, Reza Yousefi-Nooraie, Martin S. Zand, Deborah J. Ossip

**Affiliations:** 1 Department of Neurology, University of Rochester Medical Center, Rochester, NY, USA; 2 Center for Leading Innovation and Collaboration (CLIC), Clinical and Translational Science Award Program National Coordinating Center, University of Rochester Medical Center, Rochester, NY, USA; 3 University of Rochester Clinical and Translational Science Institute, University of Rochester Medical Center, Rochester, NY, USA; 4 Kennedy Krieger Institute, Baltimore, MD, USA; 5 Department of Public Health Sciences, University of Rochester Medical Center, Rochester, NY, USA; 6 Currently with the Center for Biologics Evaluation and Research, Food and Drug Administration, Silver Spring, MD, USA; 7 Department of Medicine – Division of Nephrology, University of Rochester Medical Center, Rochester, NY, USA

**Keywords:** Team science, unconference, Un-Meeting, collaboration, concept mapping, opioids, translational science

## Abstract

Advances in translational science require innovative solutions, and engagement of productive transdisciplinary teams play a critical role. While various forms of scientific meetings have long provided venues for sharing scientific findings and generating new collaborations, many conferences lack opportunities for active discussions. We describe the use of an Un-Meeting to foster innovative translational science teams through engaged discussions across multidisciplinary groups addressing a shared theme. The Un-Meeting was delivered by the University of Rochester Center for Leading Innovation and Collaboration, the national coordinating center for the National Institutes of Health Clinical and Translational Science Awards (CTSA) program. This pilot CTSA program Un-Meeting focused on engaging translational scientists, policy-makers, community members, advocates, and public health professionals to address the opioid crisis. The participant-driven format leveraged lightning talks, attendee-led idea generation, and extensive breakout discussions to foster multidisciplinary networking. Results indicated participation by a broad set of attendees and a high level of networking during the meeting. These results, coupled with the growth of the Un-Meeting across the CTSA Consortium, provide practices and models to potentially advance team and translational science. While future work will further assess the impact of Un-Meetings, this format presents a promising approach to enhance translational science.

## Un-Meetings in the context of scientific exchange

The scientific meeting has been a fundamental activity of researchers for centuries. Such gatherings provide a venue for presentation of new ideas, discussion of scientific findings, professional and social interactions, and opportunities to forge new collaborations. Since Frances Bacon established the meetings of the Royal Society of London in 1660, scientists have highly valued informal and unstructured “hallway conversations,” which often lead to collaborations, new research, and even lifelong friendships [1–3]. Over the last several decades, however, the format of many scientific conferences has included an increasing proportion of activities with a static format, such as extended sessions of multiple oral presentations from individual speakers, each punctuated by only a few short minutes for questions. This format emphasizes one-way transfer of information from the lecturer to an audience, with only brief interactive discussion.

**Figure 1.**
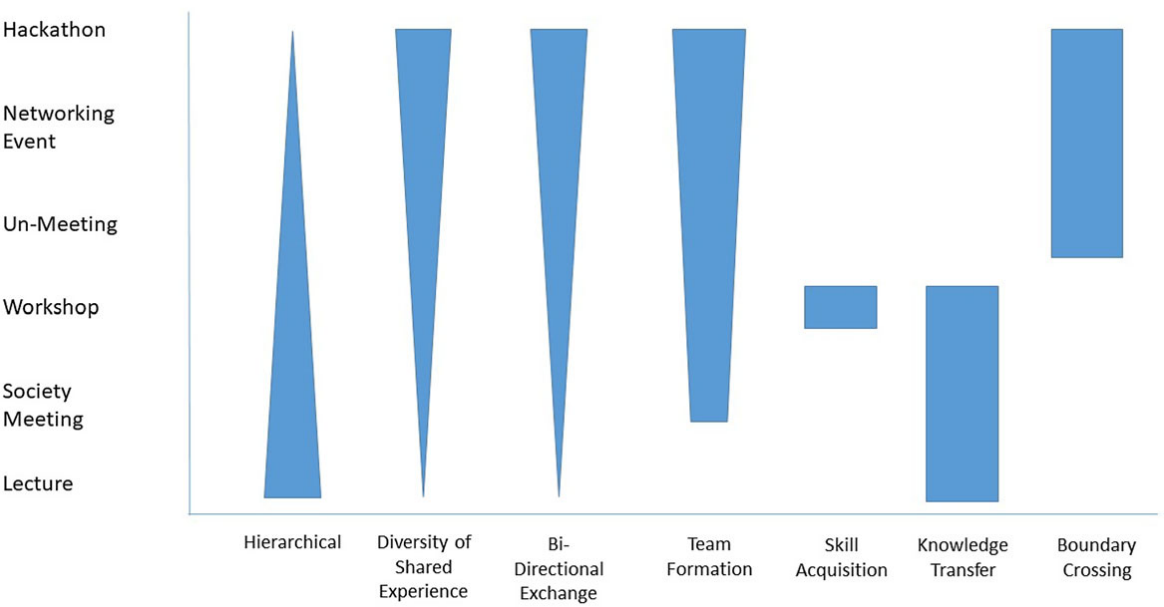
Conceptual model of the collaborative nature of meeting engagement based on meeting structure. Meeting formats that range from limited (single lecture/grand rounds) to extensive (hackathon) levels of engagement.

In 1828, Alexander von Humboldt sponsored what was perhaps the first scientific meeting intentionally structured to encourage scientists to gather in small, cross-disciplinary groups for interactive discussions [4,5]. Recently, a similar conference style has emerged in the technology sector: the “Un-Meeting” or “Unconference” [6,7]. One of the first modern Un-Meetings, known as Foo Camp, was held in 2003 [5]. In contrast to the structure of typical scientific conferences (Fig. 1), Un-Meetings maximize informal, transdisciplinary, and collegial conversations while minimizing the unidirectional presentation format [8–11]. Generally, Un-Meetings are organized around a broad topic (e.g., big data in healthcare, gene editing, and clinical implementation science), and the meeting agenda and content are created in real time by attendees. Un-Meetings are specifically structured to emphasize one-on-one or small-group in-person interactions and typically aim for fewer than 150 attendees to facilitate such an environment. Prior work has found that the Un-Meeting format facilitates in-depth conversations on a specific topic of interest, enables mutual learning across disparate scientific fields, fosters connectivity, and generates increased professional network connections and future collaborations [12–15].

Multiple studies have demonstrated the value of working in boundary-spanning or cross-disciplinary teams, including higher productivity, higher impact, and greater innovation [16]. A recent examination of key characteristics of a successful translational scientist highlights the importance of team science approaches, collaboration across research fields, integration of disciplinary silos, and ability to communicate and engage with diverse stakeholder groups [17]. The Un-Meeting format may be particularly advantageous in translational science, where transdisciplinary teams are critical to move from discovery to clinical adoption using insights from multiple fields [17,18].

Accelerating this process by fostering the development of transdisciplinary teams is one of the major goals of the National Institutes of Health (NIH) Clinical and Translational Science Awards (CTSA) program [19]. Thus, developing new methods to spark novel and effective collaborations among translational scientists is a key goal, making the Un-Meeting format a highly relevant tool for the CTSA Program Consortium.

In this report, we present the first Un-Meeting engaging the entire CTSA Program Consortium, highlight key elements and best practices to consider for Un-Meetings, discuss how the Un-Meeting model can be a tool for innovation and team formation within translational science, and suggest future research to test hypotheses regarding the outcomes of Un-Meetings.

## Key elements for translational science Un-Meetings

### Meeting theme selection and overall format

The Center for Leading Innovation and Collaboration (CLIC), housed at the University of Rochester, was the national coordinating center for the CTSA program through June 2023. CLIC hosted the first pilot of a CTSA-wide Un-Meeting on June 2, 2018 addressing the opioid crisis. The choice of topic was part of the priority response of the CTSA Program to the March 2017 United States Presidential Executive Order Establishing the President’s Commission on Combating Drug Addiction and the Opioid Crisis as well as the ongoing work of the NIH National Center for Advancing Translational Sciences (NCATS) and initiatives in the CTSA Program Consortium [20–24]. The CLIC organizing team modeled the event after similar “un” formatted events such as Health Camp Foundation’s annual unconference and the University of Rochester’s Center for Health Informatics’ Healthcare Deep Data Dive [25]. A CLIC guide for Un-Meeting planning is available [26].

### Meeting planning

Un-Meeting planning was initiated with selection of a multidisciplinary Steering Committee of government, academic, and community experts in the field of opioid use and team science. This group provided feedback on overarching goals for the Un-Meeting, identifying presenters, and assisting in the development of the agenda and the Un-Meeting structure. They also served as thought leaders, generating initial topic areas and helping to identify key participants from across a range of fields and sectors. As this meeting format was new to many, including the Steering Committee and invited speakers, planning included education on the Un-Meeting format and goals for participation to facilitate creation of the desired environment.

### Promotion and registration

Open registration was promoted throughout the CTSA Program Consortium and distributed broadly to academic institutions and organizations working on opioid-related issues. There was no registration fee, and travel stipends were provided to 27 individuals from CTSA Program hubs (awardee sites) nationally. The Un-Meeting was hosted at the University of Rochester, in a setting that provided a variety of spaces, including an open atrium to promote gathering and spontaneous interactions, an auditorium for a small number of very brief presentations, and small conference rooms for breakout sessions.

### Un-Meeting format

This Un-Meeting focused on engaging translational scientists, policy-makers, community members, advocates, and public health professionals from a variety of disciplines and backgrounds to address the opioid crisis. The event began with an informal reception the night before the Un-Meeting to initiate the process of networking and open discussion in preparation for the following day’s events.

On the day of the Un-Meeting, an “Un-Agenda” (Table S1), based loosely on the overview of the rules of an Un-Conference by Budd *et al*., provided a framework for emergent interactions and conversations [8]. The Un-Meeting began with an introduction to the objective of collaboration and the “Un-Rules” (Table 1), which served as an opportunity to define Un-Meeting goals, provide an explanation of the format, and set the stage for open, interactive dialog throughout the day. Next, a series of lightning talks (“4 × 4s”; 4 minutes, 4 slides) provided a high-level overview of current research initiatives and information related to opioids, and a sense of potential topics for the subsequent small group breakout sessions. The 4 × 4 presenters included individuals from the local Monroe County Department of Health, NIH, US Department of Veteran’s Affairs, academic researchers, and a foundation director working in the field of opioid use disorder. Four presentations were given in the morning and four in the afternoon, with each set occurring prior to the ideation and breakout sessions described below.

**Table 1.** Un-rules

Un-rule	Description
The Law of Mobility	By design, Un-Meetings are very fluid and flexible in nature. Attendees are free to go where interests lie and leave if interest wanes. Similarly, potential collaborators may want to start working on ideas right away, claiming a place to chat with coffee.
The Law of Curiosity	No one knows everything … just ask! There are NO wrong questions … or answers. This goes for acronyms and jargon too. Feel free to “translate in real time” when more explanation about specific terms is needed.
The Law of Efficiency	We want to make every second count. The day is in your hands, and we are open to opportunities for efficiency. Feel free to start jotting down ideas on Post-Its early. Or, when your breakout group meets, start talking right away. If no one has started the discussion yet, feel free to jump in.
The Law of Flow	Be open to however the Un-Meeting might unfold. Whatever happens … happens. Whoever comes are the right people. Whatever happens is the only thing that could have. Whenever it starts is the right time. When it’s over, it’s over.
The Law of Momentum	The Un-Meeting is as much about what happens after the event, as it is about the event itself. We hope you’ll pledge at least one action item as you leave. We want to continue the conversation. Together, we can turn possibility into reality
The Law of Making Space	Strive to let all voices be heard. Each individual has valuable knowledge, experience, and contributions to bring to the table. Be cognizant of your own style of communicating and flex if possible. Do you usually speak up first? Try waiting an extra moment. Do you usually spend most of the time listening? Try speaking up earlier. As a group, we want to have the utmost respect, consideration, and time for all viewpoints.

### Topic generation for breakout sessions

**Figure 2.**
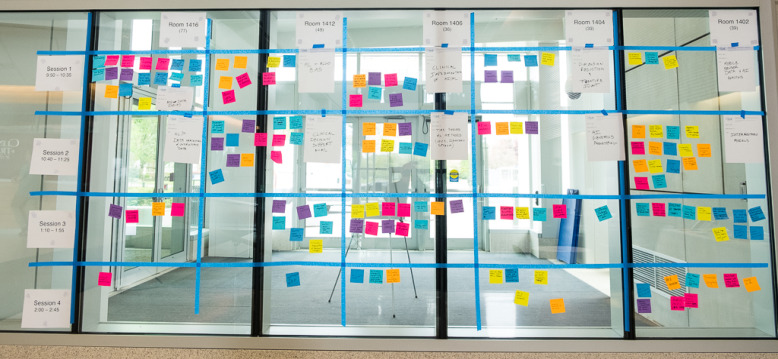
Idea generation board – A photograph of the idea generation board. Rows represent time slots, columns represent room locations for the breakout sessions, with each box enclosing the suggestions for the breakout session theme. Movable sticky notes contain concepts and ideas for the breakout session themes and are generated and grouped dynamically by the attendees throughout the Un-Meeting. Prior to each set of breakout sessions, an overarching title is given to the session, which is announced to the attendees.

An idea generation board served as a central focal point for the Un-Meeting and was used to identify topics for attendee-generated breakout sessions. After each round of 4 × 4 presentations, attendees were invited to brainstorm, write their ideas for breakout sessions on sticky notes, and place them within a grid of breakout session times on a large glass window in the atrium (Fig. 2). The Un-Meeting organizing team grouped the ideas into common themes and then aggregated the themes into two sets of concurrent breakout sessions in the morning and completed this process again following the afternoon 4x4 session. The overall number of topics and breakout sessions (23 in total, Table 2) was driven directly by participant input; attendees freely selected which sessions to attend.

**Table 2.** Breakout sessions created from topic generation

	Morning breakout topics	Afternoon breakout topics
Set 1	Rural Communities Criminal Justice Clinical Trials Pediatric and Adolescence Best Practices/Late Stage Translation/Quality and Outcomes Data Informatics	Recovery and Resilience Research Role of the CTSA Infectious Diseases and Opioids Strategies for Reducing Overdose Deaths Disparities in Addiction, Access to Treatment Prescribing Approaches
Set 2	Rx in Primary Care Community Engagement Pregnancy/Maternal/Neonatal/Child Chronic Pain, Trauma, Suicide, Risk Prevention Special Populations Non-Pharmacological Complementary Alternative Medicine	Detox Centers Community Outreach / Training Mobile / Health Technology Pharmacological Interventions Health Insurance/Policy

### Breakout session format

The 45-minute breakout sessions each had 8 to 25 attendees. Though we did not assign formal moderator roles, natural moderators emerged, and CLIC team members distributed themselves across sessions to assist as needed. Breakout rooms were arranged so that participants sat facing each other across a rectangular arrangement of tables, with easy access to an exit if the attendee wished to move to another session. These breakout sessions facilitated thematic discussions among stakeholders from a variety of backgrounds and levels of experience.

### Closing session

The Un-Meeting concluded with a brief closing session that presented a high-level snapshot of key points, open comments from the attendees, and a review of current funding opportunities for related research.

### Considerations for measuring and analyzing outcomes

Our analytical goals for the Un-Meeting were grouped into primary and secondary outcomes. The primary goals included examining the extent to which the Un-Meeting reached a broad range of attendees and fostered networking (defined as making new connections and planning further research collaborations/projects). Secondary outcomes included an exploratory evaluation of longer-term team science-related activities of grant submissions and manuscript collaboration at a 6-month follow-up.

### Attendee attributes

At the time of registration, we collected attendee demographics, including organizational affiliation, academic or professional title, career stage, field/discipline, and geographic location. Attendees included 102 individuals (97 were non-CLIC attendees) from 40 institutions, agencies, and organizations, including 27% of attendees who were affiliated with a CTSA hub (Table 3). A broad spectrum of academic career stages were represented. Attendees represented 30 different fields/disciplines (note: multiple designations could occur). Clinical fields/disciplines included: Mental Health/Psychiatry/Psychology *N* = 20; Addiction/Substance Use *N* = 16; Primary Care *N* = 7; Pain Medicine *N* = 6; Pediatrics *N* = 4; Chiropractic/Physical Therapy/Non-pharmacological treatment *N* = 3; and others *N* ≤ 2: Anesthesiology, Cardiology, Dermatology, Emergency Medicine, Hepatology, Immunology, Nursing, Nutrition, Oncology, Nutrition, Pharmacy. Nonclinical disciplines/fields included: Data Analytics/Informatics/Epidemiology *N* = 9; Public Health *N* = 8; Clinical and Translational Science *N* = 6; Biology/Neuroscience *N* = 5; Health Services *N* = 4; Social/Behavioral/Implementation Science *N* = 4; and others *N* ≤ 2 each: Administration/Quality Improvement, Communication, Education, Law/Criminal Justice. In addition, attendees were from geographically dispersed locations, with 70% of attendees coming from outside of a 100-mile radius of the Un-Meeting location and reflecting 16 other US states and the District of Columbia.

**Table 3.** Attendee attributes (N = 102)

Category		*n* (%)
Organization type	Academic	76 (74.5%)
	NIH	3 (2.9%)
	Other government agency	11 (10.8%)
	Community organization	5 (4.9%)
	Foundation	1 (1.0%)
	Other	6 (5.9%)
Academic status	Assistant professor	23 (22.5%)
	Associate professor	23 (22.5%)
	Professor	21 (20.5%)
	Residents, fellows, postdoctoral fellows	5 (5%)
Discipline^1^	Clinical	68 (66.7%)
	Nonclinical	43 (42.2%)

1 Total exceeds *N* = 102 and 100% because some attendees listed both clinical and nonclinical disciplines.

### Networking assessment

At the end of the Un-Meeting and at a 6-month follow-up time point, participants were surveyed to assess their perspectives and experience of the Un-Meeting environment and outcomes in order to assess networking activities and to provide an exploratory look at short- and longer-term outcomes (Tables S2 and S3). Relevant to the current report, the post-meeting survey included an overall evaluation of the Un-Meeting and networking, operationalized as formation of new connections, generation of new research ideas, and action planning (e.g., planned joint manuscript, grant proposal, and other research). This post-meeting survey was available at the conclusion of the Un-Meeting via a QR code provided at the location. Two reminder emails were sent to all attendees within 2 weeks of the event. Survey data were collected using the REDCap® platform.

A REDCap® link for a 6-month follow-up survey was emailed to attendees, followed by two reminder emails to non-responders. In addition to three demographic questions, the survey included three closed-ended and three open-ended questions about impacts of Un-Meeting participation on respondents’ current work and/or collaborations (e.g., joint manuscripts and grant applications). Open-ended items were coded iteratively, via open and then axial coding of each quote or passage. Text responses were independently coded by a minimum of two coders, facilitating emergence of domain-based themes from the data. Themes that emerged were further organized within broader domains and then compared among and between domain areas as described previously [27].

### Survey results

A total of 40 responses (of 97 non-CLIC attendees; 41.2%) were received from the post-Un-Meeting survey representing 28 CTSA Program hubs. Respondents positively rated the overall event and networking activities. In total, 90.0% of respondents (*n* = 36) agreed/strongly agreed that there was adequate networking time, and 95.0% (*n* = 38) made new connections at the Un-Meeting, of whom 86.8% (*n* = 33) indicating new connections made were with individuals from fields other than their own. Of note, 77.5% (*n* = 31) of respondents indicated they developed new research ideas during the meeting. Of these, 54.8% (*n* = 17) identified next steps for research, and of these, 41.2% (*n* = 7) indicated they planned to develop grant submissions, 23.5% (*n* = 4) indicated they would develop joint manuscripts, 17.6% (*n* = 3) indicated they would add or potentially add clinical trial sites, and 41.2% (*n* = 7) indicated other action steps that they planned to take after the Un-Meeting (e.g., conduct feasibility study, contact potential collaborators, and create institutional working group) [28].

For the 6-month post-Un-Meeting follow-up, 26 survey responses (26.8% of 97 non-CLIC attendees) were received, representing 25 CTSA Program hubs. Two respondents reported actively collaborating on a joint manuscript or funding opportunity as a result of the Un-Meeting. Sixty-five percent (*n* = 17) agreed that attending the Un-Meeting continues to impact their work. Two themes emerged from the open-ended items. The most common theme was “understanding and learning new ideas” (*n* = 13) in response to the question of other benefits and outcomes of the Un-Meeting. One individual commented, “the greatest benefit was hearing how physicians and mid-levels were searching for solutions to the opioid crisis. Many diverse ideas were explored throughout the Un-Meeting.” A second theme (*n* = 9) was identifying potential collaborators, with comments including “I developed a number of in-person connections that will hopefully develop into a future collaboration,” “I really liked the format and it created a nice opportunity for networking,” and “I met one of my best new collaborators there.”

## Advancing team science

### Expansion of the Un-Meeting model

CLIC has continued to host and support hubs in hosting a total of 11 Un-Meetings focusing on a broad range of translational science topics [29]. As of June, 2022, topics (in addition to addressing the opioid epidemic [30]) included rural health and health equity [31], machine learning and artificial intelligence applications for translational science [32], lifespan and life course research, clinical research in the COVID era and beyond, the critical need for professional workforce development [33], tackling the digital divide to improve telehealth, inclusion of community hospitals in clinical trials, climate change and human health through a translational science lens, enabling and promoting inter-institutional clinical and genomic research, and making real-world data and real-world evidence a reality for translational science. During the COVID-19 pandemic, CLIC expanded to use of virtual platforms for Un-Meetings, using online polling to capture the results of ideation sessions and to rank topics on interest, and breakout rooms to help replicate small interactive portions of the Un-Meeting. CLIC has also provided advice and consultations to other CTSA hubs as they develop plans to organize Un-Meetings [33].

Additionally, there has been an expansion of this model in diverse formats. Tailored and modular Un-Meetings have also been created to advance team science to address research needs, such as the Michigan Institute for Clinical & Health Research (MICHR) Research Jams at the University of Michigan [34]. These Jams utilize the ideation and visioning elements of the Un-Meeting model to identify innovative approaches to address a research priority and build collaborative teams to implement an action plan through pilot or other funding opportunities.

### Supporting teams following an Un-Meeting

There is a critical need to nurture teams formed during an Un-Meeting by providing mechanisms to support nascent collaborations, whether through direct funding or other forms of support. Many CTSA Program hubs and other centers and schools within an academic institution provide small pilot funding to seed research projects in the early stages that could be leveraged. Beyond existing programs, new small-scale pilot programs could be developed to specifically foster collaborative proposals from teams emerging from an Un-Meeting, aligned with an institution’s research priorities.

For Un-Meetings described here that span the CTSA Consortium, CTSA Program hubs could consider collaborative pilots that would support collaborations between investigators across two or more hubs (with hubs supporting their participants in the team). Additionally, the NCATS CTSA Program Collaborative Innovation Awards Program is well suited to support these types of translational team science projects, bringing together two or more CTSA Program hubs [35].

Beyond direct funding, other types of support for meeting planning and manuscript development can help to continue initial engagement and concept development that occurs during an Un-Meeting. As one example, CLIC has provided logistical and other support for CTSA Program hubs to develop Synergy Papers, with calls for Synergy Papers aligned with Un-Meetings hosted at the CLIC University of Rochester site and encouraged for Un-Meetings supported by CLIC at other hub sites [30–33].

The Synergy Paper is a collaborative manuscript developed by three or more CTSA Program hubs spanning at least two stages of translational research, with the goal of addressing substantial challenges in clinical or translational research [36]. In the context of an Un-Meeting, this can also be well suited for teams considering an innovative approach to address a significant policy or operational issue that represents a significant barrier to advancing translational science. Establishing a CTSA Discussion Forum or Special Interest Group through the Association for Clinical and Translational Science can provide other mechanisms to allow a team to continue with organized meetings and maintain the initial momentum to plan future initiatives.

### Un-Meetings as a model for providing insight into team formation

Developing methods to measure the evolution of ideas and professional network formation within Un-Meetings holds the potential to more rigorously study the earliest stages of scientific team formation and collaboration. During an Un-Meeting, the agenda for breakout sessions is generated by asking participants to place notes containing topics of interest on a grid, and then having participants and facilitators rearrange and aggregate these notes into aligned session topics. This dynamic process has similarities to concept mapping [37]. In addition, it is similar to the formation of transdisciplinary teams, requiring a convergence of ideas from disparate scientific disciplines [38]. Thus, this format lends itself to the application of network science and semantic analysis of Un-Meetings to potentially improve our understanding of transdisciplinary team formation. Other work is underway to analyze this process using mixed quantitative and qualitative methods [39].

## Discussion

Un-Meetings are designed to foster new and innovative thinking through networking and engaged discussions across diverse groups that are addressing a common issue. This first CLIC led, CTSA-wide Un-Meeting succeeded in achieving its primary goals of engaging a heterogeneous group of attendees, fostering networking across fields, and generating new research ideas, thus demonstrating the viability of the Un-Meeting concept for the CTSA program.

Attendees included a broad range of participants from hubs funded by CTSA Program and hub affiliates, which represent geographically diverse areas of the USA, a broad spectrum of the translational science continuum, and a variety of disciplines, other organizations, and stakeholders. Attendance and interaction by a range of participant professional levels, from students to experts across a variety of fields, was a key objective and results indicated that this occurred. Such diversity may facilitate cross-disciplinary team formation. Indeed, attendees reported formation of new, cross-disciplinary collaborations and relationships with the potential for future scientific team formation. Exploratory examination of longer-term impact suggested at least two resulting manuscripts/grant proposal preparation collaborations, in addition to a CLIC-supported associated Opioid Synergy Paper [30].

CLIC continued to support Un-Meetings in varying geographic locations, and with travel grants, to enhance geographic diversity of attendees and create locally based opportunities for engagement of internal and external stakeholders who might not otherwise attend [31]. This included experimenting with innovative virtual formats to replicate the Un-Meeting model.

Whether the Un-Meeting format produces greater interaction, networking, and cross-field, cross-translational stage interaction relative to traditional meetings remains to be tested. To our knowledge, no such study currently exists. Given the needs of translational science for transdisciplinary team formation, this seems promising for future research. Studying the types of contacts established at academic conferences or professional society meetings versus Un-Meetings would increase the understanding of scientific communities and may also encourage practical improvements for conference organization [40]. In addition, though beyond the scope of the current paper, bibliometric and subsequent grant analyses linking Un-Meeting attendees on topics related to the opioid crisis could examine change in the trajectory of these indicators, as well as subsequent collaborations, as potential longer-term metrics of impact for future Un-Meetings.

We note that our ability to measure interactions and networking in this report was dependent on attendee self-reports. Additional data collection using new and existing technologies for objective measurement of these variables (e.g., electronic proximity monitoring) is planned for future work and may enhance the robustness of results. Finally, the current implementation demonstrated a high level of networking and potential collaborative team formation by the end of the Un-Meeting. Though based on a small number of respondents, results of the 6-month evaluation indicated two teams moving forward with grant proposals and publications, and other respondents indicated continued collaboration and impact on their work. This suggests a potentially promising effect of the Un-Meeting on longer-term outcomes. Connecting teams with other opportunities within and outside of the CTSA Program for further work (including CTSA Enterprise Committees and Working Groups) linking Un-Meetings with deliverable-oriented infrastructures, pilot funding opportunities, and other innovative team science solutions may serve to maintain the forward momentum established at the Un-Meetings. Adding more robust methods for examining downstream results of such initiatives can better model the impact of Un-Meetings.

Overall, the CLIC Un-Meeting pilot addressing the opioid epidemic demonstrates the feasibility and opportunity for use of this interactive, nontraditional meeting format to engage a diverse group of cross-disciplinary, cross-translational stage attendees to address a critical area in translational science.

## Supporting information

Augustine et al. supplementary material Augustine et al. supplementary material

## Supplementary material

The supplementary material for this article can be found at https://doi.org/10.1017/cts.2023.576.
