# Supplementary material for: Utilizing the Un-Meeting model to advance innovative translational and team science
Source: J Clin Transl Sci. 2023 Jul 10;7(1):e176. doi: 10.1017/cts.2023.576 (PMC10514683; doi:10.1017/cts.2023.576)

**Utilizing the Un-Meeting Model to Advance Innovative Translational and Team Science**

**Supplementary Data**

Table S 1 **- Un-Agenda**

|  | **Activity** |
| --- | --- |
| **7:45 a.m.** | Registration / breakfast / networking |
| **8:30 a.m.** | Welcome and opening remarks  Martin Zand MD, PhD – Center for Leading Innovation and Collaboration (CLIC) |
| **8:40 a.m.** | Framing the issues - 4 slides x 4 minutes (4x4’s)   - Michael Kurilla, MD, PhD, NCATS - Addressing the Opioid Epidemics Through Translational Science: The CTSA Program Role - Redonna Chandler, PhD, NIDA - The Opioid Crisis: When Evidence Based Interventions Fail to Reach Patients - Ken Leonard, PhD, University at Buffalo - The Elder Side of the Epidemic: High and Increasing Death Rates in Older Adults - Christian Thrasher, Clinton Foundation - Providing the Tools and Resources to Ensure Success with Long Term Recovery |
| **9:00 a.m.** | Idea generation & room topic discussion identification |
| **9:45 a.m.** | Morning breakout session 1 |
| **10:30 a.m.** | Morning breakout session 2 |
| **11:30 a.m.** | Lunch / networking |
| **12:20 p.m.** | Re-framing the issues - 4 slides x 4 minutes (4x4’s)   - George Mashour, MD, PhD, University of Michigan - Reducing Opioid Prescribing and Enhancing Disposal: A Preventive Approach - Paul Dougherty, DC, DABCO, Canandaigua VA Medical Center - Whole Health Initiative, the Role of Patient Centered Non-Pharmacological Pain Management - Michael Mendoza, MD, MPH, MS, FAAFP, Monroe County Health Department, University of Rochester - Responding to the Opioid Epidemic in Monroe County: A Cross-Sector Collaborative Approach |
| **12:40 p.m.** | Idea generation & room topic discussion identification |
| **1:00 p.m.** | Afternoon breakout session 1 |
| **1:45 p.m.** | Afternoon breakout session 2 |
| **2:45 p.m.** | Closing remarks |

Table S 2 **– Un-Meeting Post-Meeting Survey**


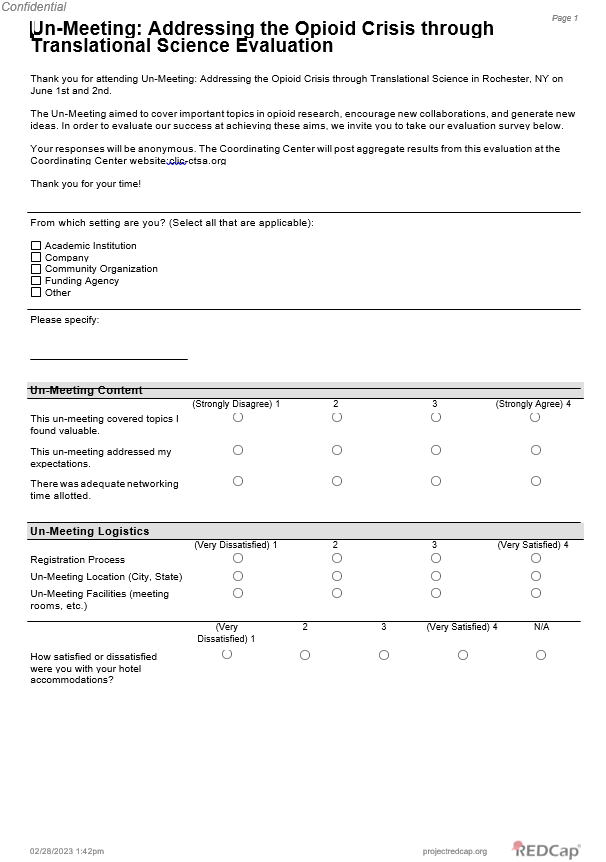


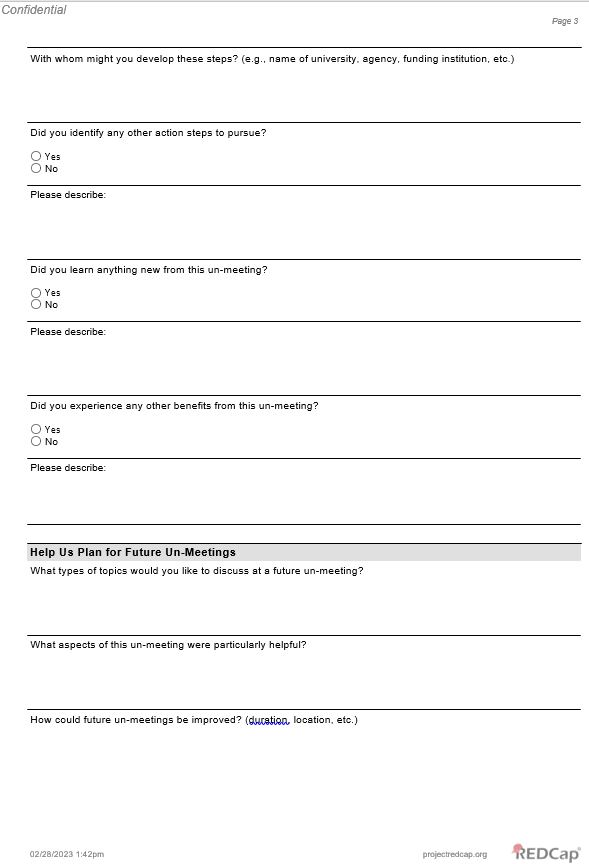


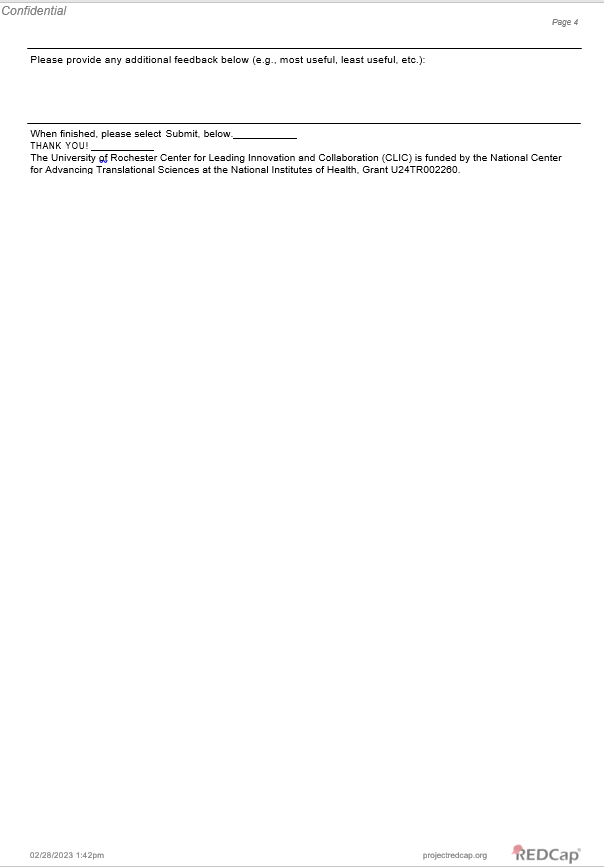


Table S 3 **– Un-Meeting 6-Month Follow-up Survey**


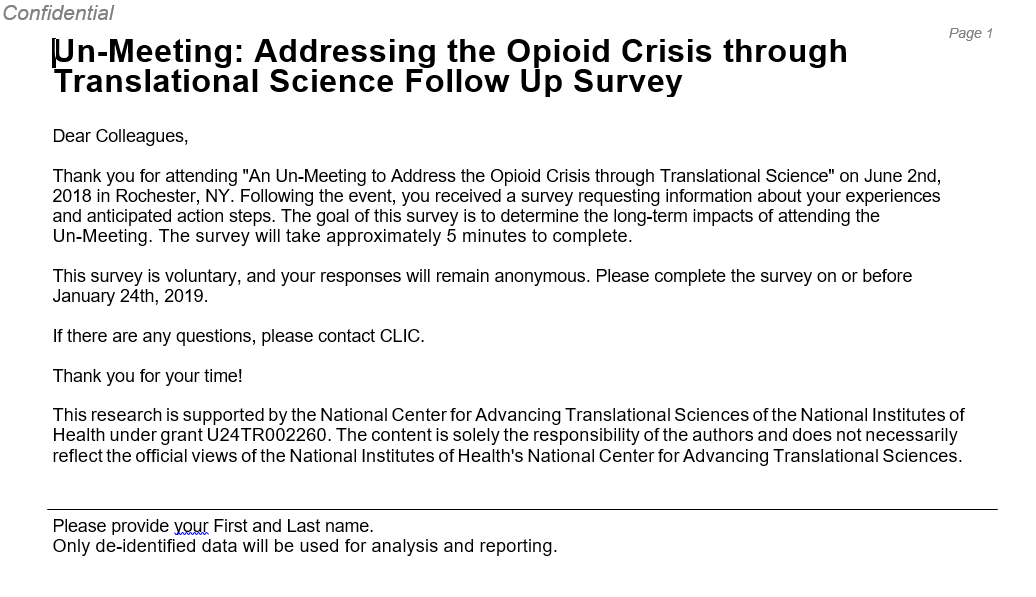


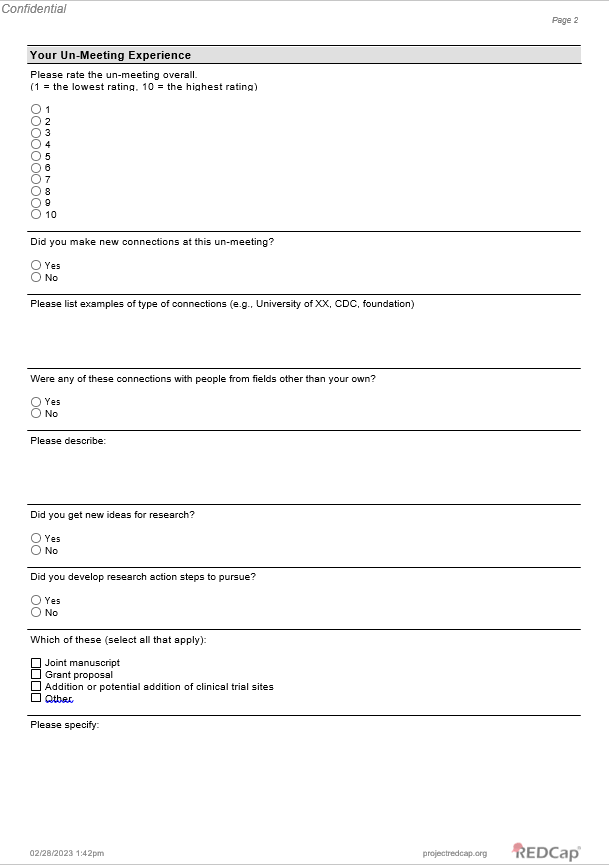


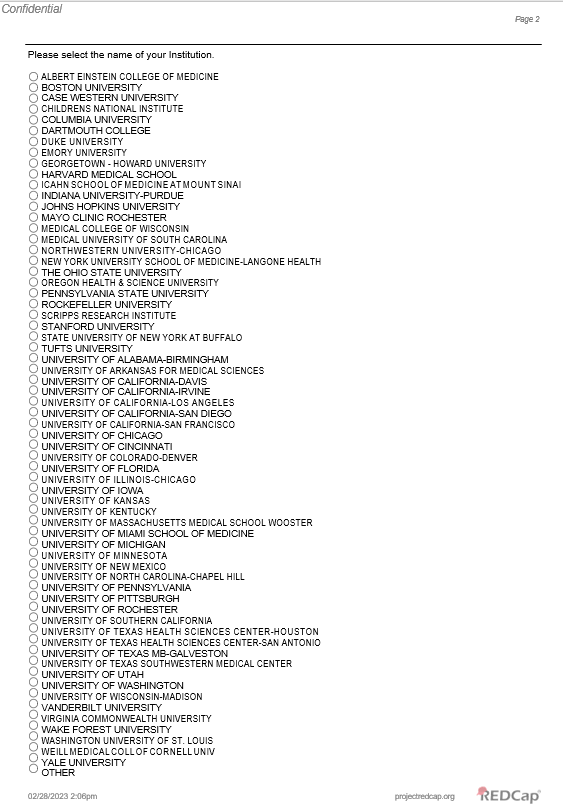


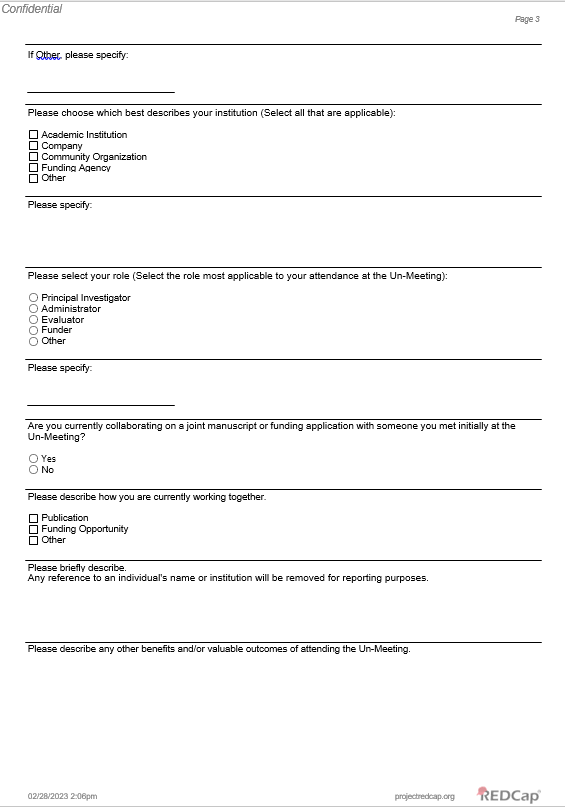


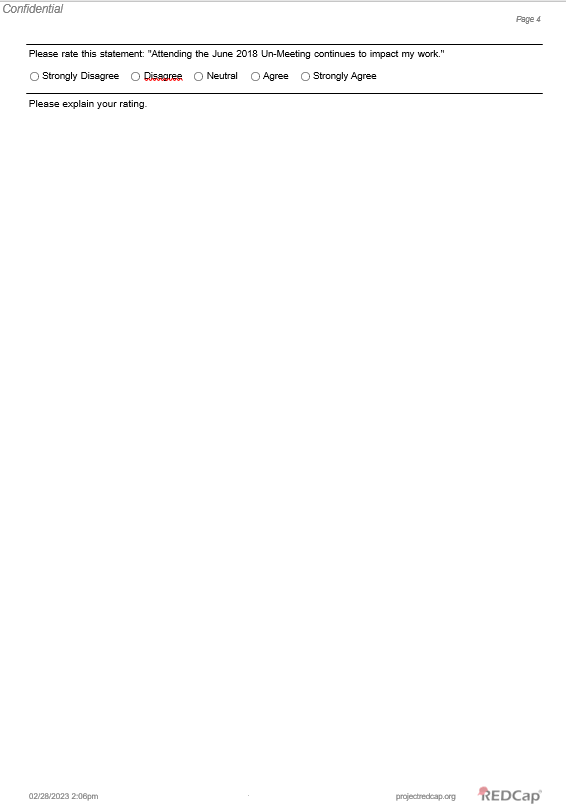

Supplement: Augustine et al. supplementary material [file S2059866123005769sup001.docx]
